# Interrogating Mitochondrial Biology and Disease Using CRISPR/Cas9 Gene Editing

**DOI:** 10.3390/genes12101604

**Published:** 2021-10-12

**Authors:** Jia-Xin Tang, Angela Pyle, Robert W. Taylor, Monika Oláhová

**Affiliations:** 1 Wellcome Centre for Mitochondrial Research, Translational and Clinical Research Institute, Newcastle University, Newcastle upon Tyne NE2 4HH, UK; j.x.tang2@ncl.ac.uk (J.-X.T.); angela.pyle@ncl.ac.uk (A.P.); robert.taylor@ncl.ac.uk (R.W.T.); 2 NHS Highly Specialised Service for Rare Mitochondrial Disorders, Newcastle upon Tyne Hospitals NHS Foundation Trust, Newcastle upon Tyne NE2 4HH, UK

**Keywords:** CRISPR/Cas9, genome editing, genome-wide CRISPR libraries screening, mitochondrial disease, cell and animal models, mitochondrial biology

## Abstract

Mitochondrial disease originates from genetic changes that impact human bodily functions by disrupting the mitochondrial oxidative phosphorylation system. MitoCarta is a curated and published inventory that sheds light on the mitochondrial proteome, but the function of some mitochondrially-localised proteins remains poorly characterised. Consequently, various gene editing systems have been employed to uncover the involvement of these proteins in mitochondrial biology and disease. CRISPR/Cas9 is an efficient, versatile, and highly accurate genome editing tool that was first introduced over a decade ago and has since become an indispensable tool for targeted genetic manipulation in biological research. The broad spectrum of CRISPR/Cas9 applications serves as an attractive and tractable system to study genes and pathways that are essential for the regulation and maintenance of mitochondrial health. It has opened possibilities of generating reliable cell and animal models of human disease, and with further exploitation of the technology, large-scale genomic screenings have uncovered a wealth of fundamental mechanistic insights. In this review, we describe the applications of CRISPR/Cas9 system as a genome editing tool to uncover new insights into pathomechanisms of mitochondrial diseases and/or biological processes involved in mitochondrial function.

## 1. Introduction

The Clustered Regularly Interspaced Short Palindromic Repeats (CRISPR)/Cas9 technology derived from bacterial adaptive immune response system, was adapted for mammalian genome editing almost a decade ago [1]. It is composed of two main components, a Cas9 endonuclease that mediates DNA cleavage and a single guide RNA (sgRNA) composed of a CRISPR RNA (crRNA) that defines the genomic target and a trans-activating CRISPR RNA (tracrRNA) that forms the scaffold for crRNA and Cas9. The sgRNA molecule directs Cas9 to the targeted genomic locus for the induction of double strand breaks (DSB) to initiate a DNA repair process, which can be exploited for genome editing [2]. In order for Cas9 to function, a short sequence (~2–6 nucleotides) named the protospacer adjacent motif (PAM) is essential for Cas9 cleavage activity and target recognition [3]. The subsequent repair of DSBs can occur via two different pathways. The predominant non-homologous end-joining (NHEJ) mechanism is error-prone and can introduce random mutations at the DSB sites resulting in deletions, insertions or frameshift mutations [4]. Alternatively, the DSBs can be repaired through homology-directed repair (HDR), which is more precise and specific and requires the presence of a customised homologous DNA repair template [5]. Depending on the context of application, both NHEJ and HDR repair mechanisms can be harnessed by the CRISPR/Cas9 system for gene editing. One of the most frequently used CRISPR/Cas9 applications is to generate (i) gene knock-outs (CRISPR-KO) to permanently disrupt gene expression of the targeted gene [1] and (ii) knock-ins (CRISPR-KI), where the DSB can be utilised for specific modifications by the HDR mechanism; however, this requires the introduction of a donor template alongside the CRISPR/Cas9 system to facilitate the knock-in of the specific change at the targeted genomic site [6]. 

Further advancements of the CRISPR/Cas9 system allowed for the construction of a catalytically deactivated Cas9 protein to recruit transcriptional inhibitors or activators to the targeted gene, termed as CRISPR inhibition (CRISPRi) and activation (CRISPRa) [7,8,9,10,11,12,13]. More recently, the application of CRISPR/Cas9 in three-dimensional genome organisation (CRISPR-GO) was established. This enabled control of targeted genomic DNA in different nuclear compartments and thus it offers a platform to investigate the correlation between genomic loci positioning and gene expression [14]. Additionally, the CRISPR/Cas9 system can be employed for reversible epigenetic regulation for gene silencing (CRISPRoff) by mediating DNA methylation, which can be reversed by CRISPRon, consisting of demethylases and transcriptional activators [15]. Due to its high accuracy in gene targeting, CRISPR/Cas9 fused with fluorescent proteins or oligonucleotides have also been successfully applied in live cell imaging for DNA and RNA visualisation [16,17,18].

Early applications of CRISPR/Cas9 technology focused on specific gene edits, but soon the system became multiplexed and it has been utilised as a powerful tool for large-scale unbiased screening using genome-wide CRISPR libraries. Two different formats of CRISPR libraries have been developed—pooled and arrayed—enabling high-throughput genotypic and phenotypic screening. CRISPR screening libraries have been used to knock-out (loss-of-function CRISPR-KO), activate (gain-of-function CRISPRa), or inhibit (transcriptional repression CRISPRi) target genes [10,19,20,21,22]. These approaches allow extensive interrogation of genetic factors involved in various biological pathways and mapping interacting networks between the genes of interest to delineate unknown molecular mechanisms associated with specific cellular phenotypes. To date, genome-wide pooled CRISPR screens have been mainly performed in cancer cell lines, but the application of CRISPR libraries to human-derived-induced pluripotent stem cells (iPSCs) has an immense potential to reveal molecular mechanisms and/or drug targets in various diseases including mitochondrial disease.

## 2. Gene Editing in Mitochondrial Research

Mitochondria have a central role in cellular energy metabolism owing to their oxidative phosphorylation (OXPHOS) function to generate adenosine triphosphate (ATP). Due to its archaebacterial ancestral origin, mitochondria have their own multicopy mitochondrial genome (mtDNA) that encodes 37 genes, specifically 13 mRNAs, 2 rRNAs, and 22 tRNAs. Although, mitochondria have their own gene expression system to produce the 13 essential subunits for the OXPHOS machinery, they are largely dependent on the nuclear genome (nDNA) for the majority of the constituent and regulatory proteins involved [23]. With the ongoing effort to establish the MitoCarta list of mitochondrially-localised proteins to supplement current understanding of mitochondrial biology, the function of many mitochondrial proteins is still unknown or poorly characterised, thus posing further challenges in the pursuit of mitochondrial biology-related studies [24]. Previously, protein–protein interaction mapping studies uncovered a group of uncharacterised mitochondrial proteins or “orphan” proteins, and successfully assigned C17ORF89 protein as a mitochondrial complex I assembly factor and LYRM5 as a protein responsible for the deflavination of electron-transferring flavoproteins. Additionally, a group of mitochondrial ‘’orphan’’ proteins has been identified to function in a collective complex for the biosynthesis of coenzyme Q. Altogether, these studies exhibit a concerted effort within the mitochondrial research community to dissect the involvement of uncharacterised mitochondrial proteins, with a mutual goal to reduce the obstacles present in the pursuit of understanding mitochondrial biology and disease [25].

Defects in mtDNA and nDNA encoding for mitochondrial genes give rise to the manifestation of mitochondrial disease often characterised by impaired OXPHOS function [26]. The multicopy nature of mtDNA means that heteroplasmy may occur where there is a mixture of wild type and mutant mtDNA within an individual cell. Henceforth, mitochondrial dysfunctions only arise when the mutant mtDNA load exceeds a certain threshold [27]. Besides that, a large number of genetic variants causing mitochondrial pathology lie within the nuclear genome encoding for mitochondrially-localised proteins. With the genetic pathogenesis of mitochondrial disease, gene editing tools become particularly applicable in mitochondrial research to dissect complex molecular disease mechanisms.

The modification of the mitochondrial genome has its challenges, but over the years several protein-based mtDNA editing methods have been successful. For instance, mitochondrial targeted restriction endonucleases (MitoREs) were the first to be used to bind mutant mtDNA sequences containing restriction sites and induce DSBs. DSBs in mtDNA lead to the degradation of mutant mtDNA, as mitochondria lack the DSB repair mechanisms, hence facilitating the elimination of mutant mtDNA and the recovery of wild type copy number [28,29]. However, the application of MitoREs is limited because it can only be used for mtDNA sequences containing specific restriction sites for the bacterial restriction endonucleases. Other systems developed for mitochondrial genome editing include the exploitation of sequence-specific endonuclease systems, namely mitochondrial-targeted zinc finger nucleases (mitoZFNs) and transcription activator-like effector nucleases (mitoTALENs). In order to facilitate the elimination of targeted mutant mtDNA, the systems are fused with flanking mitochondrial-targeting sequence (MTS). mitoZFNs and mitoTALENs are each designed to bind as pair-recognizing specific sequences at opposite strands in close proximity to the targeted DNA. [30,31,32,33,34,35]. Both of these systems combine their modular DNA-binding domains with endonuclease domains from the *FokI* restriction enzyme, which upon dimmerisation creates a DSB in mtDNA. More recently, Zekonyte and colleagues had shown effective application of the *I-CreI* meganuclease-based ARCUS gene editing platform, successfully shifting mtDNA heteroplasmy in a wide range of tissues to nearly wild type levels, except those in the central nervous system due to limitations of adeno-associated virus (AAV9) delivery. The mitoARCUS system is advantageous due to its smaller size and homodimeric nature, which ease vector packaging, as well as due to its high specificity with no nuclear off-target edits observed in tissues harvested from mice model tested compared to mitoZFNs and mitoTALENs [36]. 

CRISPR/Cas9 technology has proven to be highly effective in nuclear genome editing, hence being widely applied for genetic manipulation in the understanding of mitochondrial biology and disease, which will be discussed in the following chapters. However, the mechanism of nucleic acid import into mitochondria remains elusive, and therefore, leaving the feasibility of the CRISPR/Cas9 system for mitochondrial gene editing debatable [37]. A breakthrough in genome editing using a new DNA base-editor approach that adapts some of the CRISPR concepts of gene editing became an exciting new tool to facilitate precise manipulation of the mitochondrial genome. The base editing system consists of a catalytically inactive Cas9 protein conjugated to a bacterial deaminase. It is guided by a sgRNA to facilitate single-nucleotide conversion via deamination without the requirement of DSBs, donor DNA template, or the HDR [38,39,40]. To exploit its use in modifying the mtDNA, a CRISPR-free mitochondrial base editor—DddA-derived cytosine base editors (DdCBEs) adapted from the toxin domain of a bacterial cytidine deaminase (DddAtox) was established. It is delivered by a bacterial intracellular protein transport mechanism called Type VI secretory system. To avoid cell toxicity, DddAtox was split into two halves fused with mitochondrial targeting signal (MTS)-linked TALE array proteins and an uracil glycosylase inhibitor (UGI) to only be reassembled as active DdCBEs at the targeted mitochondrial gene sites. DdCBEs were targeted at six mitochondrial genes in HEK293T cells (*MT-ND1, MT-ND2, MT-ND4, MT-ND5, MT-ND6, MT-ATP8*) and were able to catalyse C•G-to-T•A conversions at variable efficiencies (5–50%) without inducing DSBs, therefore minimising the risk of unnecessary off-target editing [41]. The CRISPR-free mtDNA base editor is a promising new tool to create precision models of mitochondrial disease. Although, the current approach is limited to certain nucleotide conversions, it is likely that other base editing systems will be discovered in the near future to expand the editing capacity of mtDNA.

## 3. Application of CRISPR/Cas9 in Mitochondrial Research

**Figure 1:**
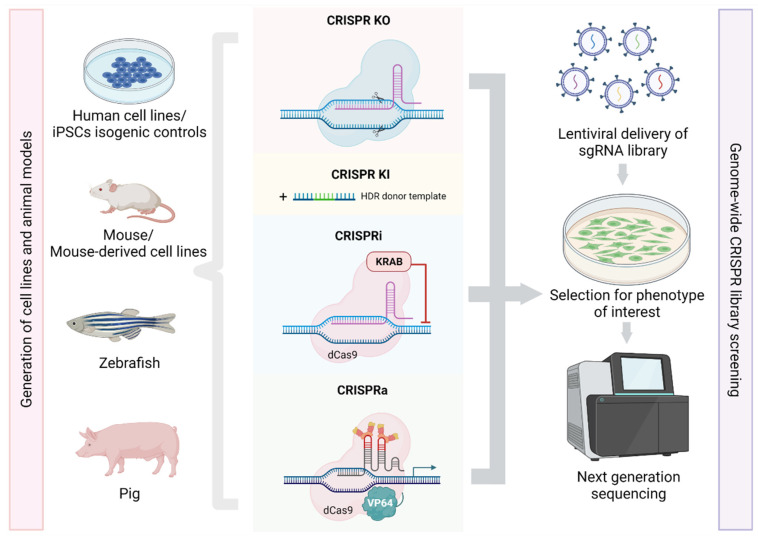
Implementation of CRISPR/Cas9 technologies in biomedical research. The CRISPR/Cas9 system is a highly accurate and efficient genome editing tool that has been exploited for gene knockout (CRISPR-KO), gene knock-in (CRISPR-KI), and transcriptional inhibition (CRISPRi) or activation (CRISPRa) utilising the catalytically inactive dCas9 and the e.g., repressor KRAB or activator VP64 domain, respectively. These methods have been taken advantage of to generate reliable cell and animal models, to dissect basic mitochondrial functions and pathomechanisms. CRISPR/Cas9 gene editing has been also widely used to generate isogenic controls from human-derived iPSCs, thus minimising experimental variability due to different genetic backgrounds. A recent avenue of CRISPR/Cas9 technology is genome-wide CRISPR screening to uncover new genetic factors under specific selection pressure. The sgRNA library is usually delivered into cell lines *via* lentiviral particles, and following the selection process, the cell population is sequenced using next generation sequencing platforms and the top hits are identified. Created with Biorender.com (accessed on 15 September 2021).

The versatility of CRISPR/Cas9 technology enables researchers to exploit different aspects of mitochondrial biology and disease. As mentioned earlier, mitochondrial research has been challenging due to the presence of a large number of mitochondrial orphan proteins without functional annotation. Since different approaches have been employed to understand the function of these poorly characterized proteins through the study of genetic variants implicated in mitochondrial disease patients, in addition to various ‘omics’ and CRISPR gene editing methods, which will be discussed in the following section (Figure 1).

### 3.1. Generation of Cell Lines and Animal Models

CRISPR/Cas9 technology offers a reliable approach to generate cell lines and animal models to study the function of different gene products in mitochondrial biology and disease. The use of these will be described below, highlighting three main areas of applications:

1. Delineating specific gene functions to understand basic mitochondrial molecular mechanisms;
2. Generating cell lines and animal models to establish and examine gene causality for mitochondrial dysfunction;
3. Generating isogenic control cell lines (i) to provide reliable controls minimising experimental variability and (ii) to verify pathogenicity of mitochondrial disease-causing candidates

#### 3.1.1. Delineating Gene Functions in Mitochondrial Biology

A member of the NOL1/NOP2/Sun (NSUN) family of RNA methyltransferases NSUN2, was previously known to be involved in post-transcriptional modification of cytoplasmic tRNAs by cytosine-5 methylation (m5C); however, recently it has also been established as a m5C modifier of mitochondrial tRNAs (mt-tRNAs). The researchers confirmed the mitochondrial localisation of NSUN2 in human cells and a general decrease of m5C signals to mt-tRNAs in both *Nsun2^−/−^* null mice and patient fibroblasts harbouring homozygous *NSUN2* splice mutation. To support these findings, an *NSUN2* CRISPR/Cas9 knockout (KO) in U-2 osteosarcoma (U2-OS) cell line was generated and the impact of loss of NSUN2 in m5C of mt-tRNAs was determined. This resulted in a reduction in m5C signals specifically in the variable loop region containing cytosine at the positions 48 to 50 in mt-tRNAs, which was partially restored by the re-expression of mitochondrial-targeted NSUN2 [42]. These data strengthened the evidence for NSUN2 involvement in m5C modification of mt-tRNA variable loop region [42,43,44,45].

CRISPR/Cas9-mediated genetic manipulation can also be applicable in animal model generation, which in conjunction with edited human cell lines, can delineate the correlation of specific mitochondrial proteins and mitochondrial pathology both in vitro and in vivo. As an example, YARS2—a mitochondrial aminoacyl-tRNA synthetase—has recently been investigated using HeLa cell lines and a zebrafish model to characterise its pathological role in the presence of defective copies of the gene. CRISPR/Cas9 *YARS2* KO cell lines showed aberrant mt-tRNA aminoacylation, leading to impaired mitochondrial bioenergetics affecting OXPHOS biogenesis and stability, in addition to compromised mitochondrial proteostasis. The severe OXPHOS defects were also observed in zebrafish models of *yars2^−/−^* CRISPR/Cas9 KO. Furthermore, the zebrafish models exhibited tissue-specific effects of the loss of YARS2 protein in the retina, due to photoreceptor degeneration that leads to visual impairment—a phenotype also observed in patients harbouring *YARS2* mutation. The mt-tRNA aminoacylation defect in both the KO cell line and zebrafish models were effectively rescued by the delivery of a wild type copy of the *YARS2* gene, confirming its pathological role in mitochondrial bioenergetics problems [46].

#### 3.1.2. Generation of Cell Lines and Animal Models to Examine Gene Causality for Mitochondrial Dysfunction

As patient-derived biopsies can often be scarce and limiting in terms of producing replicate results, CRISPR/Cas9-generated cell and/or animal models are useful when determining pathogenicity and the underlying pathological mechanism(s) of a mitochondrial disease gene. For instance, *Ndufs4^−/−^* (encoding for mitochondrial complex I subunit) KO mice have been created by a one-step procedure, co-injecting the Cas9 mRNA and sgRNA targeting *Ndufs4* gene directly into the pronuclei of the zygotes. This is a more cost- and time-saving method to generate germline modified mice compared to traditional methods. The *Ndufs4^−/−^* KO mice mimic the human Leigh syndrome, which is normally caused by severe mitochondrial dysfunction, most commonly due to mitochondrial complex I deficiency. Importantly, this study confirmed the role of NDFUS4 in early embryonic development and successfully recapitulated Leigh-like physical and behavioural characteristics in mice, thus providing a useful animal model to study the molecular mechanisms underlying complex I deficiency in Leigh syndrome patients [47].

Pathogenic variants in the mitochondrial tRNA-Specific 2-Thiouridylase 1, *Mtu1* gene have been associated with liver failure and hearing disabilities in humans [48,49,50]. To demonstrate the importance of Mtu1 in mitochondrial health, a *mtu1^−/−^* KO zebrafish model was generated using CRISPR/Cas9 technology. The *mtu1^−/−^* KO in zebrafish verified its impact in the post-transcriptional modification of mt-tRNAs at the wobble position, leading to decreased levels of mitochondrial OXPHOS proteins. Most importantly, the zebrafish model demonstrated the hearing defects, likely due to isolated mitochondrial defects in the hair cells of the inner ear resulting from the loss of Mtu1 protein, thus signifying its pathological association with deafness in humans [51].

Animal models harbouring genetic changes in *SURF1*, the gene encoding the mitochondrial complex IV assembly factor is a well-established model of mitochondrial disease (mice, zebrafish and flies). *SURF1* defects account for a considerable proportion of Leigh syndrome cases associated with cytochrome *c* oxidase (COX) deficiencies [52,53,54]. While these animal models provide evidence of COX deficiency due to loss of SURF1 protein, they were unable to recapitulate the majority of clinical features in *SURF1* Leigh syndrome patients. Furthermore, *Surf1^−/−^* KO mice present with extended lifespan. Therefore, to study the impact of pathogenic genes on disease presentation and progression, it is important to generate animal models of species that share anatomical and physiological similarities to humans. To address this issue, the first pig model of mitochondrial disease carrying *SURF1* mutations was created. Firstly, *SURF1* was disrupted in pig fibroblasts cell lines using CRISPR/Cas9, to be used in the generation of heterozygous and homozygous *SURF1* KO pigs through somatic cell nuclear transfer. Thus, generated *SURF* pigs were then characterised for their clinical and biochemical phenotypes. Despite discrepancy in the OXPHOS enzyme activities across different tissue types when compared to Leigh syndrome patients, the *SURF1* KO pigs managed to replicate the neuropathology in patients that was not observed in *Surf1^–/–^* mouse models. This eludes the pathogenesis of *SURF1* in mitochondrial respiratory metabolism, particularly relating to neuromuscular symptoms in the patients [55].

The CRISPR/Cas9 system also enables interrogations on tissue-specific level to examine distinct effects of mitochondrial proteins. A study investigating the mitochondrial role of the pentatricopeptide repeat domain protein 1 (PTCD1) employed CRISPR/Cas9 to generate *Ptcd1^−/−^* conditional KO mice models, as complete loss of PTCD1 causes embryonic lethality. The conditional KO strategy involved the introduction of a HDR DNA template spanning across exons 3 to 5 of the gene, which was modified to add *LoxP* sites to exon 3 and move two bases from exon 4 up to exon 3 to enable frameshift without affecting mRNA splicing. The *Ptcd1*^loxP/loxP^ mice were then crossed with Cre-recombinase-expressing mice regulated by muscle creatinine kinase promoter to produce heart- and skeletal muscle-specific *Ptcd1^−/−^* KO mice. Data from this study established a new tissue-specific role for PTCD1 in mitoribosome assembly by stabilising the mitochondrial 16S rRNA—an essential factor for maintenance of normal mitochondrial gene expression [56].

#### 3.1.3. Generating Isogenic Control Cell Lines to Verify Pathogenicity of Mitochondrial Proteins

Nevertheless, the application of CRISPR/Cas9 is not limited to generation of physiological models, but is also useful in creating isogenic iPSC controls for modelling mitochondrial diseases. Isogenic controls are more advantageous than healthy or diseased control cell lines as they represent a homogenous population of cells with defined genotype. Hence, they eliminate the possibility of background variations, which enhances robustness of the differences between patients and controls. Patient-derived iPSCs are widely applied in modelling of human diseases, including mitochondrial disease as they can be differentiated into relevant cell types to study distinct mitochondrial disease phenotypes [57]. For instance, a study of Charcot-Marie-Tooth Type 2 (CMT2) disease generated *CMT-2* patient-derived iPSCs to characterise CMT2 disease phenotypes in neuronal network. CMT2 is a type of mitochondrial disorder that causes peripheral neuropathy by affecting axonal structure and function. Importantly, this study generated isogenic control cell lines with the corrected *CMT2* mutation using CRISPR/Cas9. These control and patient iPSCs were differentiated into different neuronal subtypes and the group was able to demonstrate that impaired axonal transport in iPSC-derived motor neurons are likely linked to mitochondrial energy deficits, which was restored in the CRISPR/Cas9 corrected isogenic control cell line [58]. Another example is a study that generated iPSCs from a patient harbouring *CoQ4* mutation causing coenzyme Q10 deficiency and utilised CRISPR/Cas9 to edit and correct the mutations to obtain isogenic controls [59].

### 3.2. High-Throughput CRISPR/Cas9 Library Screening

The development of genome-wide CRISPR/Cas9 screening libraries has allowed exploration of mitochondrial research questions in a high-throughput and unbiased approach. In particular, pooled CRISPR/Cas9 screens enabled time and cost-effective interrogation of the genome to narrow down candidate genes that confer phenotype of interest and more importantly the potential of delineating the interplay between genetic factors in biological pathways. Screening selections can be divided into two categories (i) negative screens to identify genes essential for survival or growth (cells that did not survive the selection condition) and (ii) positive screens to identify cells that survived the selective pressure (e.g., drug), which needs to be robust to narrow down the final number of hit genes. In response to a specific condition, a marker gene expression system to select for cells that express phenotype of interest can be employed. Such screens encompass a wide array of phenotypic measurements, such as growth, fluorescence-activated cell sorting or imaging of cellular phenotypes [22]. High-throughput next generation sequencing and analysis platforms before and after the selection will determine the depletion of sgRNAs in a negative screen and enrichment in a positive screen. 

The first genome-wide CRISPR/Cas9 screen to identify genes required for mitochondrial OXPHOS function was performed by Arroyo and Jourdain, et al. The screen is referred to as “CRISPR death screen” because it positively selects for cells that express the cell death marker Annexin V when grown in galactose selection conditions, as opposed to identifying gene depletion, which is more challenging. OXPHOS deficient cells grown in glucose-rich media predominantly rely on glycolysis rather than OXPHOS for ATP production. However, replacing the media with galactose induces a metabolic shift towards oxidative metabolism. Stringent parameters were defined for the screen to ensure only knocked-out genes that cause galactose-specific cell deaths (essential OXPHOS genes) were shortlisted. This was done by eliminating genes that are likely broadly lethal and those that confer growth defects in galactose-rich media. This experiment resulted in the identification of 300 hits with a threshold false discovery rate (FDR) of 30% and 191 hits with high confidence (FDR < 10%). Over 30% of the 300 hits do not have a homolog in *Saccharomyces cerevisiae*, which can be vastly different from human mitochondria, but widely used to study mitochondrial biology. The death screen also revealed 229 OXPHOS-essential genes that were either not previously linked to the disease or reported in the MitoCarta 2.0 list of mitochondria-localised proteins. An important output of a genome-wide CRISPR/Cas9 library screen is the follow up analysis of the enriched genetic hits. This identified new genetic pathways important for the maintenance of the mitochondrial protein synthesis machinery, including a functional module of genes essential for mitochondrial 16S rRNA regulation and mitochondrial translation [60].

Another genome-wide CRISPRi library screening identified essential genes that maintain ATP levels through OXPHOS only, by measuring real-time ATP levels using fluorescence resonance energy transfer (FRET)-based fluorescence activated cell sorting. The high-throughput CRISPRi screen targeting 2231 genes in a mitochondrial gene-enriched library verified the importance of many genes in the event of ATP deficiency, in particular mitochondrial ribosomal proteins and the CoQ10 biosynthesis machinery. Interestingly, a number of hits from the screen that are involved in the CoQ10 pathway were observed to rescue the ATP depletion upon supplementation with CoQ10, thus providing gene-specific protection against ATP deficits. Over 60% of the OXPHOS essential genes identified from the death screen conducted by Arroyo and Jourdain, et al. were also identified in this study. However, half of the hits from the screen were not reported by the death screen. It is likely that genes involved in ATP maintenance might not necessarily be essential for survival by OXPHOS. In addition, some cells with defective OXPHOS function could potentially survive and escape the death screen. These studies demonstrate the strength of large-scale CRISPR library screening, which can reveal the role of previously unknown genes, when two different methods of phenotypic selection are employed. Altogether, these studies improve our understanding of genetic and metabolic interactions in the regulation of mitochondrial energy metabolism [61].

As described above, besides revealing essential genes for mitochondrial function, genome-wide CRISPR/Cas9 screening is also applicable in delineating a variety of mitochondria-related cellular processes. Mitophagy is a cellular program that targets damaged mitochondria for degradation to maintain mitochondrial and cellular homeostasis. A genome-wide CRISPR/Cas9 knockout screen was carried out in mouse myoblasts with Parkin overexpression, which mediates the predominant mitophagy pathway. Mitophagy was induced in mouse myoblasts by exposure to mitochondrial stressors, carbonyl cyanide m-chlorophenyl hydrazine (CCCP), or the OAR cocktail of inhibitors (oligomycin inhibiting complex V, antimycin A inhibiting complex III, and rotenone inhibiting complex I). The study aimed to uncover genetic components that drive Parkin-mediated mitophagy through flow cytometry that detects fluorescence signals representing mitophagy activity in the cells. Adenine nucleotide translocator (ANT) complex appeared to be an essential regulator of the mitophagy process, and this function was independent of its known role in nucleotide transport across mitochondrial membranes. This has been verified in *Ant1* knockout mouse model and patient cardiac tissue with homozygous loss-of-function *ANT1* mutation, which manifested phenotypes relating to defective mitophagy and mitochondrial quality control [62]. 

The integrated stress response (ISR) pathway is a conserved stress signalling network, which activates an ATF4-dependent survival gene expression programme. Mitochondrial dysfunction activates the ISR [63]; however, the underlying molecular mechanism of mitochondrial stress signalling was unknown, until Guo and colleagues identified HRI as the Eukaryotic Translation Initiation Factor 2A (eIF2α) kinase that induces ATF4 expression upon mitochondrial stress. To define upstream components that lead to ATF4 activation via HRI, the group performed a large-scale CRISPR interference (CRISPRi) screen targeting 7710 protein coding genes using a catalytically inactive Cas9 protein (dCas9) fused to a transcriptional repressor, Kruppel-associated box (KRAB) domain. The screen revealed *HRI* and *DELE1* as the top hits resulting in reduced ATF4 expression when knocked down in the event of mitochondrial stress. Further experimentation confirmed the localisation of DELE1 into mitochondrial membranes, and identified the mitochondrial protease OMA1 as an essential factor that facilitates the cleavage of DELE1, which subsequently leads to up-regulation of *ATF4* gene expression. The identification of the core signal-transducing protein, DELE1 and HRI, in the mitochondrial stress response could contribute as potential therapeutic targets for mitochondrial disease conditions that exacerbates mitochondrial stress response [64].

Genome-wide CRISPR/Cas9 screens have also been very informative in the investigation of potential therapeutic treatments using mitochondrial disease models. Mitochondrial dysfunction can be mimicked in immortalised cell lines by inhibiting mitochondrial respiratory chain components or by disrupting mitochondrial respiratory metabolism. This has been taken advantage of by a study that identified protective factors for mitochondrial OXPHOS defects through a genome-wide CRISPR knockout screen in K562 cell lines modelled for (i) moderate mitochondrial disease condition by adding complex III inhibitor, antimycin alone and (ii) severe disease conditions using antimycin and removal of pyruvate to worsen reductive stress. The screen identified Von Hippel-Laindau (VHL) factor as an important endogenous factor in conferring survival of mitochondrial-defective cells when the gene was knocked out by five different sgRNAs targeting all exons. VHL factor regulates the hypoxia response by targeting hydroxylated hypoxia-inducible transcription factors (HIFs) under normoxic conditions for degradation. As there were no suitable VHL inhibitors for delivery into cells, an alternative small molecule, FG-4592, which triggers hypoxia response, was used. FG-4592 mimics the *VHL* KO by inducing hypoxia response and was shown to rescue the growth defect caused by moderate and severe mitochondrial disease conditions in human cell lines. These findings were then validated in a zebrafish *vhl^−/−^* knockout and a mouse model of Leigh syndrome caused by disruption of the *Ndufs4* gene, encoding a subunit of mitochondrial Complex I. This study poses a possibility of hypoxia induction as mitochondrial disease therapy by potentially triggering an adaptive response mechanism(s) that decreases the body’s reliance on oxidative respiratory rate [65]. 

Genome-wide genetic screens in parallel to complement high-throughput chemical screens can be a useful tool to interrogate chemical-genetic cellular interactions. For instance, a genome-wide CRISPR/Cas9 knockout screen in human cybrids harbouring a m.3796A>G *MT-ND1* gene variant was performed to complement a high-throughput chemical screen, identifying chemical compounds that rescued the mitochondrial complex I defect. I-BET 525762A, an inhibitor of bromodomain-containing protein 4 (BRD4), was revealed as a strong candidate in the chemical screen. Importantly, BRD4 was one of the top hits identified in the CRISPR/Cas9 genome-wide KO screen that was performed under galactose growth conditions forcing cells to rely on OXPHOS for survival. Furthermore, the effect of bromodomain inhibition was verified by chemical assays employing bromodomain inhibitors and genetic ablation of *BRD4* through rescue of mitochondrial OXPHOS defects including other complex I-deficient human cybrid cell lines and patient fibroblasts. Further investigation demonstrated that bromodomain inhibition overcomes mitochondrial defects caused by complex I deficiency through dissociating BRD4 interaction at the promoters of nuclear-encoded mitochondrial genes and subsequently leading to rewiring of mitochondrial energy metabolism to exploit alternative energy sources [66].

## 4. The Use of CRISPR/Cas9 System for Targeting the Mitochondrial Genome 

Despite the broad-ranging applications of CRISPR/Cas9 technology in mitochondrial research, there are still limitations arising from the approach that obstruct its applicability in manipulating the mitochondrial genome. General limitations of CRISPR/Cas9 applications include strict PAM site requirement, off-target mutagenesis, and delivery methods into different cell types or animal models. Nevertheless, researchers have developed a variety of CRISPR components, design, and delivery approaches to make CRISPR/Cas9 more adaptable in different experimental systems. However, the delivery of the CRISPR/Cas9 system into mitochondria remains debatable and in this section, we will discuss the challenges surrounding the delivery of CRISPR/Cas9 system into the organelle.

Mitochondrial dysfunction can originate from mutations in nuclear- or mitochondrial-encoded genes and therefore manipulation of mtDNA is of great interest in efforts to delineate underlying disease mechanisms caused by high proportion of mutant mtDNA. As discussed earlier, mitochondrial-targeted restriction endonucleases, i.e., mtZFNs and mitoTALENs, have been exploited to investigate research questions relating to mtDNA heteroplasmy and successfully shifted mtDNA heteroplasmy [32,67]. However, the use of CRISPR/Cas9 technology for the manipulation of mitochondrial genome remains controversial due to poorly understood RNA import processes into mitochondria and the organelle’s different DNA repair mechanism system compared to the nucleus. Despite being studied rigorously over the years, the proposed mechanism of RNA import is still unclear due to inadequate reproducible evidence demonstrating the indispensable requirement of importing nuclear RNA into mitochondria and the definitive role of polynucleotide phosphorylase (PNPase) [37]. Furthermore, DNA DSB repair, which is the basis of CRISPR/Cas9 gene editing, was found to be lacking in mitochondria, raising concerns over the application of CRISPR/Cas9 in targeting mtDNA, although, homologous recombination-mediated repair processes were shown to be present in mitochondria [68,69].

The first study reporting mitochondrial genome editing by CRISPR/Cas9 was in 2015, in which the researchers designed a mitochondrial-targeted Cas9 by replacing the nuclear localisation signal on the N-terminus of the Cas9 protein with mitochondrial-targeting sequence (MTS). The mitochondrial-targeted Cas9 (mitoCas9) was delivered into human HEK293T cell lines together with sgRNAs targeting *MT-COX1* and *MT-COX3* and after five days of expression, a decrease in the targeted mtDNA loci was reported through RT-PCR [70]. Another report of genome editing mtDNA using the CRISPR/Cas9 system was published four years later, where the mitoCas9 protein was flanked by two MTS that were adapted from human and zebrafish *cox8a* genes. Here, Bian and colleagues reported evidence of fluorescence signals indicating mitochondrial localisation of the mito-CRISPR/Cas9 system and a single-stranded DNA (ssDNA) template to repair or introduce mtDNA mutations via homologous recombination. The mito-CRISPR/Cas9 system and ssDNA template for knock-in were tested in HEK293T cells and zebrafish embryos through delivery by microinjection. However, cleavage of the targeted mtDNA loci by the mito-CRISPR/Cas9 system was unable to be proven through T7 endonuclease 1 mismatch cleavage assay, although decreased mtDNA copy numbers and presence of loxP sites introduced to targeted sites by the ssDNA template were observed [71]. A more recent attempt to apply CRISPR/Cas9 for mitochondrial gene editing harnesses the 20-nucleotide RNAse P stem loop to form a novel sgRNA construct, which can be delivered into the mitochondria by mitochondrial RNA import protein, PNPase found in the mitochondrial intermembrane space and matrix. Here, the Cas9 protein was expressed in mitochondria by *Cas9* mRNA flanked by MTS. Cleavage of the sgRNA construct at intended mtDNA region was confirmed in mouse embryonic fibroblasts, exhibiting reduction in mtDNA levels and gene expression. If established in human cells, this modified mitochondrial-targeting CRISPR/Cas9 system could be useful in addressing a wide array of mtDNA mutations contributing to mitochondrial diseases and potentially be exploited for mitochondrial therapeutics purposes.

However, the efficiency and reliability of the mtDNA-targeting CRISPR/Cas9 system still remains controversial due to a lack of comprehensive evidence and reproducibility showing mitochondrial localisation of the CRISPR/Cas9 system, and mtDNA repair mechanism to enable precise manipulations of the mitochondrial genome. Nevertheless, despite some of their limitations, manipulation of mtDNA employing endonucleases, base editors, or meganucleases are currently a much more powerful and reliable tool for the study of mitochondrial biology and disease relating to the mitochondrial genome.

## 5. Concluding Remarks and Future Perspectives

This review highlights the applicability and challenges surrounding the use of CRISPR/Cas9 technology in mitochondrial research. With established success in employing CRISPR/Cas9 to manipulate cell lines and animal models for the interrogation of mitochondrial pathology, CRISPR/Cas9 has proven to be beneficial in producing robust and high-throughput data, which helps to delineate functional roles of previously uncharacterised mitochondrially-localised proteins. In particular, large-scale CRISPR library screening also allows the identification of potential therapeutic targets in the search of a cure for mitochondrial disease.

As mitochondrial disease encompasses a heterogenous group of disorders, it can affect various cell or tissue types, which hampers the practical application of genome editing tools in the treatment of human mitochondrial diseases. Nonetheless, several nuclease-mediated genome editing approaches, i.e., mitoZFNs, mitoTALENs, and mitoARCUS, have demonstrated robust pre-clinical evidence of cell- or tissue-specific gene targeting to correct pathogenic mtDNA variants in vivo using mouse models [34,36,72]. These genome editing tools show robust pre-clinical evidence for mtDNA heteroplasmy shifting both in vitro and in vivo, and together with the newly emerging mitochondrial base editors, are revealing promising avenues for translation into mitochondrial medicine. However, the applicability of the CRISPR/Cas9 system as gene therapy for human mitochondrial DNA disease remains a distant concept at present, as more reliable evidence of how the system is delivered into mitochondria and performs efficient genome editing is required [37]. Nevertheless, the transformative advance of CRISPR/Cas9 technology serves as a powerful approach in mitochondrial research to further explore novel aspects of mitochondrial biology, function, and the role of this fascinating organelle in disease pathology.
